# The impact of a multidisciplinary team intervention on medication prescription in nursing homes in Catalonia

**DOI:** 10.3389/fphar.2024.1445141

**Published:** 2024-09-13

**Authors:** Emilie Anderssen-Nordahl, Eladio Fernández-Liz, Mònica Sabaté Gallego, Montserrat Bosch Ferrer, Margarita Sánchez-Arcilla Rosanas, Mercè Cervera León, Joaquim Miquel Magrinyà, Maria Estrella Barceló-Colomer

**Affiliations:** ^1^ Clinical Pharmacology Service, Vall d’Hebron University Hospital, Vall d’Hebron Barcelona Hospital Campus, Barcelona, Spain; ^2^ Clinical Pharmacology Group, Vall d’Hebron Research Institute, Barcelona, Spain; ^3^ Department of Pharmacology, Therapeutics and Toxicology, Universitat Autònoma de Barcelona, Barcelona, Spain; ^4^ Primary Healthcare Barcelona, Management of Primary Care and the Community of Barcelona City, Catalan Institute of Health, Barcelona, Spain; ^5^ Foundation University Institute for Research in Primary Health Care Jordi Gol i Gurina (IDIAPJGol), Barcelona, Spain; ^6^ Geriatric Unit, Internal Medicine Service, Vall d’Hebron University Hospital, Vall d’Hebron Barcelona Hospital Campus, Barcelona, Spain

**Keywords:** drug utilization review, patient care team, frail elderly, nursing homes, potentially inappropriate medication list

## Abstract

**Background:**

In response to the rising population of nursing home residents with frailty and multimorbidity, optimizing medication safety through drug utilization review and addressing medication-related problems (MRPs) is imperative. Clinical decision support systems help reduce medication errors and detect potential MRPs, as well as medication reviews performed by a multidisciplinary team, but these combined assessments are not commonly performed. The objective of this study was to evaluate the impact on medication plans of a multidisciplinary team intervention in nursing homes, by analyzing the medication plan before and after the intervention and assessing whether the recommendations given had been implemented.

**Methods:**

A multicenter before-after study, involving five nursing homes, assessed the impact of a multidisciplinary team intervention, to estimate effectiveness related to the review of the prescribed medications. The follow-up period for each patient was 12 months or until death if prior, from July 2020 to February 2022, and involved 483 patients. The clinical pharmacologist coordinated the intervention and reviewed all the prescribed medications to make recommendations, focused on the completion of absent data, withdrawal of a drug, verification of whether a drug was adequate, the substitution of a drug, and the addition of drugs. Since the intervention was performed during the COVID-19 pandemic, optimization of psychotropic drugs and absorbent pads were limited.

**Results:**

The intervention had an impact with recommendations given for 398 (82.4%) of the patients and which were followed by 58.5% of them. At least one drug was withdrawn in 293 (60.7%) of the patients, with a mean of 2.3 (SD 1.7). As for the total of 1,097 recommendations given, 355 (32.4%) were followed. From the intervention, antipsychotics, antidepressants, benzodiazepines, statins, and diuretics were the most frequently withdrawn.

**Conclusion:**

The findings underscore the impact of targeted interventions to reduce inappropriate medications and enhance medication safety in nursing homes. The proposed recommendations given and followed show the importance of a multidisciplinary team, coordinated by a clinical pharmacologist, for a patient-centered approach to make medication reviews regularly, with the help of clinical decision support systems, to help reduce potential MRPs and polypharmacy.

## 1 Introduction

In recent years, the healthcare system has witnessed a marked rise in the number of nursing home residents with frailty and multimorbidity. It has therefore become essential to ensure that such individuals receive the safest and most accurate medication. Effective medication reviews with computerized drug utilization review (DUR) and the elimination of medication-related problems (MRPs) in nursing homes are crucial for optimizing patient care (Kojima, 2015; Fog et al., 2017; Osmani et al., 2023).

A computerized DUR is defined as a formal program for assessing drug prescription and patient safety. It assesses whether patients receive appropriate medication and aims to identify MRPs (Kim et al., 2021). Implementing DUR programs to monitor drug therapy seems to reduce the risk of medication errors and adverse drug reactions (ADRs) (Osmani et al., 2023). In primary healthcare in Catalonia, a clinical decision support system (CDSS) has been implemented to improve patient safety. It entails the Self Audit tool and PREFASEG (*PREscripción FArmacéutica SEGura*, i.e., safe pharmaceutical prescription) (Pons-Mesquida et al., 2021; Pons-Mesquida et al., 2022). A CDSS and its tools can help review patients’ medication, and should be addressed with a multidisciplinary team approach, including a clinical pharmacologist and a clinical pharmacist (Anderssen-Nordahl et al., 2024).

An MRP is a situation involving drug therapy that can potentially interfere with health outcomes. Some MRPs include therapeutic duplications, possible drug-drug interactions (DDIs), potentially inappropriate medications (PIMs), and contraindicated drugs (Troncoso-Mariño et al., 2021). It is essential to prevent MRPs through regular medication reviews to ensure the well-being of nursing home residents.

Such individuals with frailty and multimorbidity require a personalized approach to medication management and deprescribing. This involves understanding their health priorities, assessing disease burden, evaluating treatment risks and benefits, and agreeing on an individualized treatment plan (NICE Guideline, 2016). Polypharmacy and MRPs are more prevalent in this population thus increasing the risk of ADRs and DDIs (Lavan et al., 2016). Polypharmacy is defined as the simultaneous use of five or more medications, while excessive polypharmacy refers to the use of ten or more medications (Zahlan et al., 2023). Another type of inappropriate polypharmacy is the continuous addition of new drugs to manage adverse events related to avoidable medications, which can create a prescribing cascade (Falster et al., 2021). Evidence shows that the most powerful strategy to cope with inappropriate drug use and polypharmacy is poly-deprescribing, which implies stopping as many non-lifesaving medications as possible (Campins et al., 2017; Garfinkel and Bilek, 2020). Several studies have already reported that the use of deprescribing tools, supported by multidisciplinary teams with physicians, reduced inappropriate polypharmacy in hospitalized, nursing home and primary care older patients. In addition, the tools helped physicians decide whether to withdraw the prescription, how to withdraw it, and how to communicate the deprescription to older hospitalized patients (Cooper et al., 2015; Kua et al., 2019; Duong et al., 2021; Faulkner et al., 2022; Cole et al., 2023).

A multidisciplinary approach, integrating a team of healthcare professionals from different disciplines and specialties, aimed at reaching a combined decision on a complex situation, is essential for the optimal care of nursing home residents with advanced dementia. Interprofessional teamwork allows the sharing of experience, clinical expertise, varying disciplinary perspectives, and knowledge about institutionalized patients. All of which permits the performance of an effective DUR, the management of inappropriate drugs, and the creation of optimal individualized medication. Continuing with medication should be considered an active decision that carries as much responsibility as when initiating or ceasing treatment (Disalvo et al., 2020; Cole et al., 2023; Song et al., 2023). Medication reviews in Central and Eastern European countries are also conducted by clinical pharmacists. Some studies indicate that these reviews can be beneficial for the elderly, helping to prevent MRPs and ensuring the safe and effective use of medications, particularly regarding medication adherence. However, these practices remain underdeveloped and underutilized in certain parts of Europe (Ibrahim et al., 2021; Saeed et al., 2022; Urbańczyk et al., 2023). Nonetheless, in Catalonia, there is a home healthcare program (ATDOM) at the primary care level. A study intends to conduct a pragmatic randomized clinical trial with a control group to evaluate the effectiveness of a pharmacist-led intervention. This intervention will focus on optimizing the pharmacological treatment of patients enrolled in the ATDOM program. Through prospective follow-up, the study will assess the potential of the intervention to reduce MRPs and enhance the overall quality of care for these patients (Salom-Garrigues et al., 2024). Additionally, a before-and-after intervention study in Catalonia evaluated the impact of a pharmaceutical intervention on optimizing treatment for patients with type 2 diabetes mellitus. Of the recommendations made by a pharmacist or clinical pharmacologist, 54.7% were successfully implemented (Canadell-Vilarrasa et al., 2024).

Whilst many previous studies have examined the effectiveness of medicine optimization interventions to improve appropriate polypharmacy and reduce MRPs in older people and elderly individuals residing in nursing homes, there are few registered interventions of quality (Cooper et al., 2015; Saeed et al., 2022; Sluggett et al., 2022; Cole et al., 2023). As for similar interventions in nursing homes, during the SARS-CoV-2 pandemic, there are none published to date. It is estimated that 50% of medication errors and 20% of ADRs could be avoided with proper medication reconciliation, which would contribute to improving patient safety. It is therefore crucial to review and reconcile medication, carry out deprescription when appropriate, and assess adherence. According to the Catalan Health Service instruction 04/2012, all patients with chronic treatment should undergo a pharmacological review at least once a year (Department of Health, Government of Catalonia, 2014).

The SARS-CoV-2 pandemic created a great challenge for the care of institutionalized patients. For this reason, a multidisciplinary team was created in Catalonia, Spain, to perform a structured intervention in nursing homes. The intervention consisted of reviewing medication plans, detecting MRPs, and developing an improvement strategy with proposals.

The objective of this study was to evaluate the impact on medication plans of a multidisciplinary team intervention in nursing homes, by analyzing the medication plan before and after the intervention and assessing whether the recommendations proposed had been implemented.

## 2 Methods

### 2.1 Study design and setting

A multicenter before-after study was performed, without a control group, to estimate effectiveness related to the review of the prescribed medications. From a total of 48 nursing homes in the northern area of Barcelona, Spain, data were collected from 5. These 5 nursing homes were prioritized by the health administration due to their size, for efficiency, and to cover the highest population percentage. From such a selection, even though only 5 were evaluated, the intervention covered 22.3% of the total residents in the nursing homes in the northern area of Barcelona. The study population included all patients currently admitted to a nursing home at the start of this intervention, which began in July 2020. Patient follow-up was from the beginning of the intervention until 1 year later or until death if prior, finalizing in February 2022.

The inclusion criteria encompassed institutionalized patients with public health coverage provided by the Catalan Health Service during the study period. The exclusion criteria were institutionalized patients with health coverage provided by other insurers, short-term life expectancy, hospitalization during the intervention, death or discharge in the first month of the review, and individuals who could not be intervened due to lack of information. There was no formal sample size calculation since the analysis was carried at on all the reviewed patients with the exception of those excluded.

The study design, procedures, and reporting followed the TREND guidelines for nonrandomized evaluations of behavioral and public health interventions (Des Jarlais et al., 2004) and are registered at ENCePP (Reference: EUPAS106748).

### 2.2 The intervention

This structured intervention was performed during the COVID-19 pandemic. It consisted of systematically evaluating the prescribed medications, and reviewing the validity of prescriptions and medication plans. With this intervention, a description of the prescribed medication before and after a year was made, and potential MRPs were detected. The MRPs registered were potential DDIs, therapeutic duplications, contraindications, and drugs deemed inappropriate or of doubtful efficacy.

The multidisciplinary team included general practitioners (GPs), nurses, social and administrative workers from primary care, clinicians and nurses assigned to the nursing homes, a clinical pharmacist, and a clinical pharmacologist. They systematically evaluated the prescribed medications to promote safe and healthy prescription (Anderssen-Nordahl et al., 2024). The clinical pharmacologist was the medical doctor specialist who coordinated the multidisciplinary team and actively reviewed all the prescribed medications to make recommendations. These recommendations were discussed with the team and the final decision was supported or not by the physician in each nursing home, who then decided how to convey this information to the patients or their representatives. The clinical pharmacologist employed around 50 min per patient thus an average of 10 patients could be reviewed daily. Intervention duration was from the first review on 1st July 2020 to the last one on the 5th March 2021. The first follow-up after a year started on 2nd August 2021 and lasted until the final follow-up on the 28th February 2022. Since the intervention took place during the pandemic, optimization of psycholeptic drugs and absorbent pads was limited.

Several recommendations arose from the issues identified during the medication review. They included the completion of absent data, withdrawal of a drug, verification of whether a drug was adequate, the substitution of a drug, and adding a drug. With respect to the data, allergies or diseases could be absent. Drug withdrawal was recommended taking into account potential MRPs. They included potential DDIs, duplicated therapies, contraindicated drugs, inappropriate drugs, or drugs of doubtful efficacy. Adequacy of drug use was related to the need for dosage reduction, bad tolerance, lowering of the anticholinergic load, or a high risk of ADRs. As for drug substitution, this could be recommended due to considering other drugs as a first choice or an equivalent. Regarding the addition of medications, it was recommended only in specific cases: vitamin B12 and folic acid or iron for anemia and deficiency, thyroid hormone for clear hypothyroidism, osteoporotic treatment for patients with fragility fractures, and proton pump inhibitors when indicated. The addition of drugs was advised only when it was evident that they were necessary.

The standard used to establish whether drugs were considered MRPs was the information contained in the technical information sheets, the support tools Self-Audit and PREFASEG (Pons-Mesquida et al., 2021; Pons-Mesquida et al., 2022), and the list of potentially inappropriate drugs and criteria proposed by the Catalan Health Service (Department of Health, Government of Catalonia, 2014; Catalan Health Service: Department of Health, 2020).

The support tools were the Self-Audit and PREFASEG. The Self Audit identifies and systematically resolves MRPs. It generates a list of patients with active MRPs so as to facilitate treatment changes or suspensions (Pons-Mesquida et al., 2022). PREFASEG generates online notifications when starting a treatment to warn clinicians of potential problems related to drug use and prevent medication errors (Pons-Mesquida et al., 2021). The computerized medical record notifies the healthcare professionals when a patient is attended by another professional and explains the medication changes made.

The criteria proposed by the Catalan Health Service on potentially inappropriate drugs in the elderly (Catalan Health Service: Department of Health, 2020) were based on documents regarding the management of medication in chronic patients (Department of Health, Government of Catalonia, 2014). Such documents were prepared by consensus from a group of experts. The criteria for the drugs to be included on the potentially inappropriate list were to appear in at least 2 bibliographic databases, with an explicit recommendation or contraindication for the elderly population in the technical sheet, or with a specific alert from the Spanish Agency for Medicines and Health Products (AEMPS, Agencia Española de Medicamentos y Productos Sanitarios). The references used were the Beers criteria, STOPP/START, the EU-PIM list, the PRISCUS list, information notes on medicines for human use from the AEMPS, and anticholinergic risk scales in older adults (Department of Health, Government of Catalonia, 2014; Catalan Health Service: Department of Health, 2020; American Geriatrics Society Beers Criteria^®^ Update Expert Panel, 2023; Mann et al., 2023; O’Mahony et al., 2023).

**FIGURE 1:**
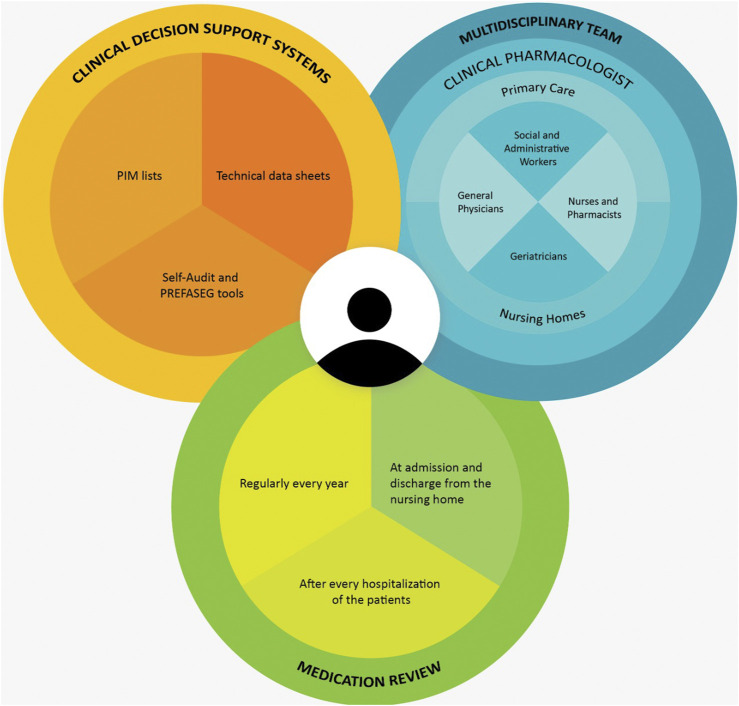
The intervention with a multidisciplinary team.

The patient-centered intervention with the multidisciplinary team, medication review, and supporting tools is shown in Figure 1.

### 2.3 Variables and data collection

The variables analyzed were the number of prescribed medications including fixed-dose combinations and absorbent pads before and after the intervention, recommendations given, drugs recommended to be withdrawn, changed or considered adequate, drugs withdrawn or added, and the number of deaths. Medications were recorded according to the Anatomical Therapeutic Chemical (ATC) classification system.

The data were collected in usual clinical practice during the intervention, from common electronic medical records. A computerized clinical history program is used by all professionals in the primary care network in Catalonia (Primary Care Clinical Station, 2024). The anonymized information was then entered into the Research Electronic Data Capture (REDCap) platform. REDCap is an electronic data capture software and workflow methodology for designing research databases for clinical trials and translational research. The privacy policies and code of conduct of REDCap platform can be consulted at the following link: https://projectredcap.org/. A quality check was carried out prior to analysis.

### 2.4 Ethics approval

The study was conducted according to the guidelines of the Declaration of Helsinki. The protocol was approved by both local Research Ethics Committees Vall Hebron University Hospital (protocol code EOM (AG) 067/2021 (5,930)) and IDIAP Jordi Gol (protocol code 22/027-P). No informed consent was necessary since the information was anonymized.

### 2.5 Statistical analysis

A descriptive analysis was performed of drugs prescribed, use of absorbent pads, recommendations given, drugs recommended to be withdrawn, changed or considered adequate, drugs withdrawn or added, and the number of deaths after a year. A comparative analysis of before and after the intervention was carried out with the total of patients, recommendations, and deaths after a year. For the analysis, continuous variables are presented as means (standard deviation, SD) and categorical variables as frequencies (percentages). Statistical analysis was performed using R version 4.3.0.

## 3 Results

### 3.1 General characteristics of the institutionalized patients

**FIGURE 2:**
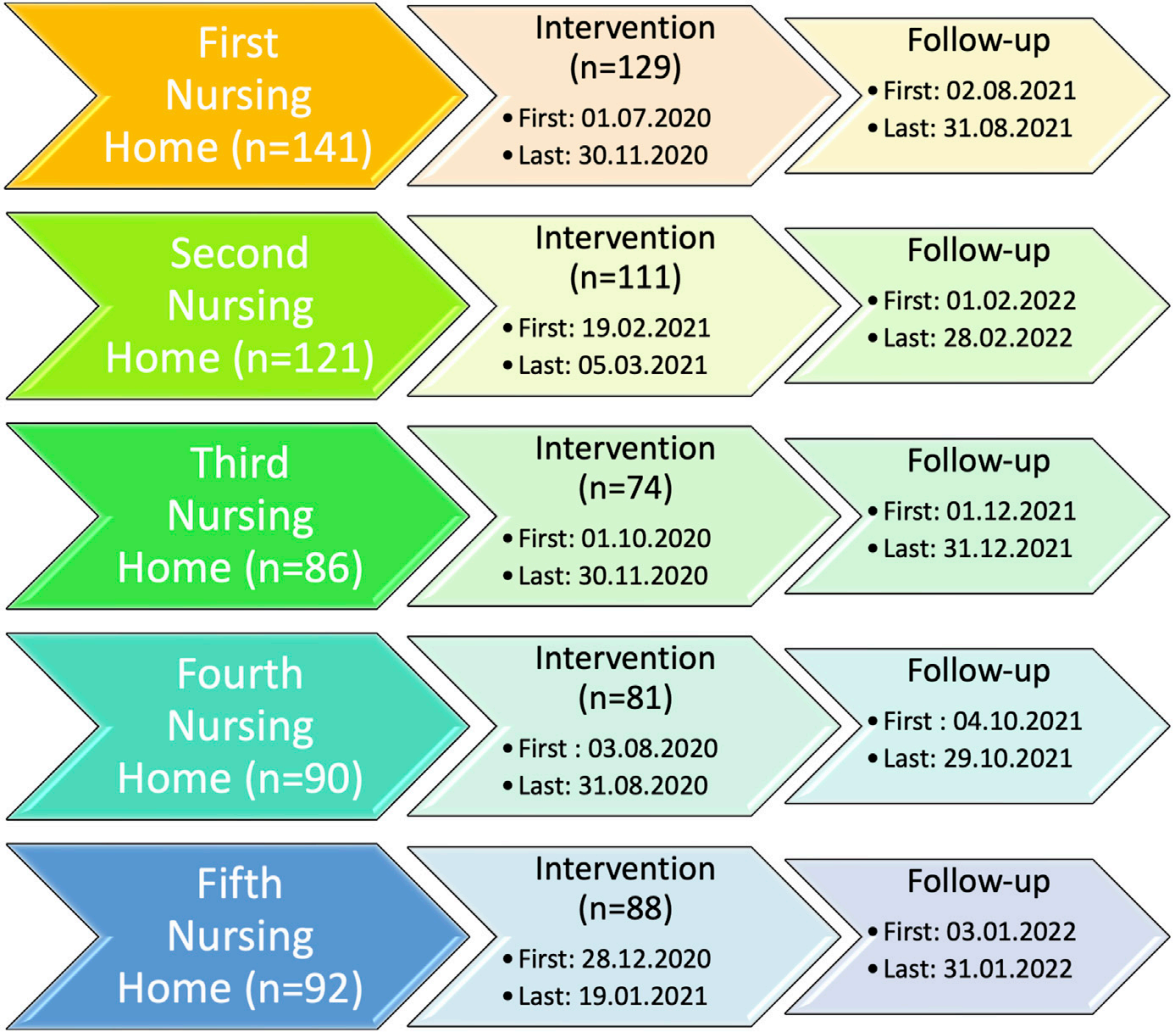
Calendar of all the patients from the intervention until follow-up divided into the five nursing homes. * All patients from the intervention were followed up.

The intervention started on 1st July 2020 and ended on 28th February 2022, with the last follow-up after a year, as shown in Figure 2.

A total of 483 patients were included from 5 different nursing homes. Initially, there were 530 patients, however, due to exclusion criteria 47 were not included. These 47 exclusions were 9 patients with health coverage provided by other insurers, 5 with a short-term life expectancy, 14 hospitalized during the intervention, 7 lost to follow-up in the first month, and 12 due to lack of information.

At baseline, the mean age of the 483 patients included was 86.3 (SD 8.8) years, and 348 (72.0%) were female. The mean of the health-related problems (HRPs) was 17.4 (SD 5.6), and the mean number of prescribed medications was 8.22 (SD 3.5), including fixed-dose combinations. All the other onset clinical characteristics, descriptive analysis of recommendations, incomplete data, medication recommended to verify adequacy of use, substitution, or withdrawal, and MRPs, have been previously described and commented on (Anderssen-Nordahl et al., 2024).

### 3.2 Impact of the intervention in nursing homes

In the 483 patients in the five nursing homes, the total number of prescribed drugs, including fixed-dose combinations, prior to the intervention and 1 year after was 3,962 and 3,893, respectively. A total of 374 (77.43%) patients used absorbent pads at the commencement of the intervention, a figure which increased to 420 (86.95%) 1 year later.

Of the 398 (82.4%) patients who received recommendations 233 (58.5%) patients followed. The recommendations given varied from 1 to 6 per patient, with a mean of 2.2 (SD 1.1). The various recommendations offered and taken up, with the total and percentage of compliance, are shown in Table 1.

**TABLE 1:** Description of all the recommendations given and followed.

	Recommendations given, n	%	Recommendations followed, n	%	%*
Completing data	173	15.8	81	22.8	46.8
Allergy data	118	10.8	66	18.6	55.9
Disease data	55	5.0	15	4.2	27.3
Withdrawal of drugs	318	29.0	136	38.3	42.8
Withdrawal of inappropriate drugs	66	6.0	35	9.9	53.0
Withdrawal of drugs with interactions	53	4.8	26	7.3	49.1
Withdrawal of duplications	33	3.0	19	5.4	57.6
Withdrawal of drugs with doubtful efficacy	22	2.0	14	3.9	63.6
Withdrawal of contraindicated drugs	16	1.5	10	2.8	62.5
Witdrawal of other drugs	128	11.7	32	9.0	25.0
Substitution of drugs	45	4.1	11	3.1	24.4
Substitution of equivalent drugs	35	3.2	8	2.3	22.9
Substitution of drug of choice	10	0.9	3	0.8	30.0
Verification of the adequacy of drug use	561	51.1	127	35.8	22.6
Total	1097	100.0	355	100.0	32.4

n = number of recommendations that were given and followed.

%*, percentage of the recommendations followed compared to those given.

A total of 318 prescribed medications were recommended to be withdrawn in 192 patients and 136 (42.8%) were removed. The five drugs most recommended in this category were omeprazole (n = 54, 17.0%), acetylsalicylic acid (n = 14, 4.4%), alprazolam (n = 11, 3.5%), simvastatin (n = 10, 3.1%), and lorazepam (n = 10, 3.1%). At follow-up, the 5 drugs that were most withdrawn were omeprazole (n = 9, 6.6%), citalopram (n = 5, 3.7%), diazepam (n = 5, 3.7%), domperidone (n = 5, 3.7%), and vitamin D and analogues (n = 5, 3.7%). All the drugs recommended to be withdrawn and those withdrawn in the pharmacological review, divided according to their ATC classification, are shown in Table 2.

**TABLE 2:** Drugs recommended to be withdrawn with the drugs withdrawn in the pharmacological review.

Drugs recommended to withdraw				Withdrawn	
		n	%	n	%*
A- Alimentary tract and metabolism					
A02BC	Proton pump inhibitors	56	17.6	9	16.1
A02BC01	Omeprazole	54	17.0	9	16.7
A02BC02	Pantoprazole	1	0.3	0	0.0
A02BC03	Lansoprazole	1	0.3	0	0.0
A03AX13	Silicones	1	0.3	1	100.0
A03FA	Propulsives	12	3.8	9	75.0
A03FA01	Metoclopramide	3	0.9	2	66.7
A03FA03	Domperidone	6	1.9	5	83.3
A03FA06	Clebopride	3	0.9	2	66.7
A05AA02	Ursodeoxycholic acid	1	0.3	0	0.0
A09AB01	Glutamic acid hydrochloride	1	0.3	0	0.0
A10B	Blood glucose lowering drugs, excluding insulins	15	4.7	2	13.3
A10BA02	Metformin	8	2.5	1	12.5
A10BB09	Gliclazide	4	1.3	0	0.0
A10BH02	Vildagliptin	1	0.3	1	100.0
A10BH03	Saxagliptin	1	0.3	0	0.0
A10BH05	Linagliptin	1	0.3	0	0.0
A11CC	Vitamin D and analogues	8	2.5	5	62.5
A12AX	Calcium with vitamin D	1	0.3	0	0.0
A12BA	Potassium	4	1.3	4	100.0
B- Blood and blood forming organs					
B01AC	Platelet aggregation inhibitors	15	4.7	1	6.7
B01AC06	Acetylsalicylic acid	14	4.4	0	0.0
B01AC23	Cilostazol	1	0.3	1	100.0
B02AA02	Tranexamic acid	1	0.3	1	100.0
B03BA	Vitamin B12 and folic acid	3	0.9	2	66.7
B03BA01	Cyanocobalamin	3	0.9	0	0.0
B05XA13	Hydrochloric acid	1	0.3	0	0.0
C- Cardiovascular system					
C01AA05	Digoxin	5	1.6	1	20.0
C01BD01	Amiodarone	1	0.3	1	100.0
C01EB15	Trimetazidine	1	0.3	1	100.0
C02CA04	Doxazosin	4	1.3	1	25.0
C02DB02	Hydralazine	1	0.3	1	100.0
C03AA03	Hydrochlorothiazide	1	0.3	0	0.0
C03CA	Sulfonamides, plain	7	2.2	3	42.9
C03CA01	Furosemide	6	1.9	2	33.3
C03CA04	Torasemide	1	0.3	1	100.0
C03DA01	Spironolactone	1	0.3	0	0.0
C04AD03	Pentoxifylline	3	0.9	2	66.7
C05AE03	Diltiazem	1	0.3	1	100.0
C05CA03	Diosmin	1	0.3	0	0.0
C07AB12	Nebivolol	1	0.3	0	0.0
C08CA01	Amlodipine	2	0.6	1	50.0
C09AA02	Enalapril	2	0.6	2	100.0
C09CA01	Losartan	1	0.3	1	100.0
C09DA07	Telmisartan and diuretics	1	0.3	0	0.0
C09DB02	Olmesartan medoxomil and amlodipine	1	0.3	1	100.0
C10A	Lipid modifying agents	17	5.3	9	52.9
C10AA01	Simvastatin	10	3.1	3	30.0
C10AA05	Atorvastatin	5	1.6	4	80.0
C10AB04	Gemfibrozil	1	0.3	1	100.0
C10AB05	Fenofibrate	1	0.3	1	100.0
D- Dermatologicals					
D01AE16	Amorolfine	1	0.3	1	100.0
G- Genito urinary system and sex hormones					
G03AC05	Megestrol	1	0.3	1	100.0
G03XC01	Raloxifene	1	0.3	1	100.0
G04BD	Drugs for urinary frequency and incontinence	7	2.2	4	57.1
G04BD08	Solifenacin	3	0.9	1	33.3
G04BD11	Fesoterodine	2	0.6	2	100.0
G04BD12	Mirabegron	2	0.6	1	50.0
G04BX01	Magnesium hydroxide	1	0.3	0	0.0
G04CA	Alpha-adrenoreceptor antagonists	3	0.9	0	0.0
G04CA01	Alfuzosin	1	0.3	0	0.0
G04CA02	Tamsulosin	2	0.6	0	0.0
G04CX01	Prunus africanae cortex	1	0.3	0	0.0
H- Systemic hormonal preparations					
H03AA01	Levothyroxine sodium	1	0.3	0	0.0
M- Musculo-skeletal system					
M01A	Anti-inflammatory and antirheumatic, non-steroids	7	2.2	6	85.7
M01AB05	Diclofenac	4	1.3	4	100.0
M01AB16	Aceclofenac	1	0.3	1	100.0
M01AE17	Dexketoprofen	1	0.3	1	100.0
M01AE52	Naproxen and esomeprazole	1	0.3	0	0.0
M04AA	Preparations inhibiting uric acid production	3	0.9	1	33.3
M04AA01	Allopurinol	2	0.6	0	0.0
M04AA03	Febuxostat	1	0.3	1	100.0
M05B	Drugs affecting bone structure and mineralization	2	0.6	1	50.0
M05BA04	Alendronic acid	1	0.3	0	0.0
M05BX04	Denosumab	1	0.3	1	100.0
N- Nervous system					
N02A	Opioids	11	3.5	4	36.4
N02AB03	Fentanyl	1	0.3	0	0.0
N02AX02	Tramadol	8	2.5	3	37.5
N02AX06	Tapentadol	2	0.6	1	50.0
N02B	Other analgesics and antipyretics	7	2.2	6	85.7
N02BB02	Metamizole sodium	4	1.3	3	75.0
N02BE01	Paracetamol	3	0.9	3	100.0
N03A	Antiepileptics	7	2.2	3	42.9
N03AE01	Clonazepam	5	1.6	2	40.0
N03AX12	Gabapentin	2	0.6	1	50.0
N04BA02	Levodopa and decarboxylase inhibitor	1	0.3	9	900.0
N05A	Antipsychotics	9	2.8	5	55.6
N05AD01	Haloperidol	4	1.3	3	75.0
N05AH04	Quetiapine	3	0.9	0	0.0
N05AL07	Levosulpiride	1	0.3	1	100.0
N05AX08	Risperidone	1	0.3	1	100.0
N05B	Anxiolytics	30	9.4	17	56.7
N05BA	Benzodiazepine derivative anxiolytics	1	0.3	1	100.0
N05BA01	Diazepam	5	1.6	5	100.0
N05BA05	Potassium clorazepate	1	0.3	1	100.0
N05BA06	Lorazepam	10	3.1	4	40.0
N05BA12	Alprazolam	11	3.5	4	36.4
N05BB01	Hydroxyzine	2	0.6	2	100.0
N05C	Hypnotics and sedatives	10	3.1	4	40.0
N05CD06	Lormetazepam	1	0.3	0	0.0
N05CD11	Loprazolam	1	0.3	1	100.0
N05CF02	Zolpidem	1	0.3	0	0.0
N05CM02	Clomethiazole	7	2.2	3	42.9
N06A	Antidepressants	22	6.9	9	40.9
N06AA09	Amitriptyline	1	0.3	1	100.0
N06AB03	Fluoxetine	1	0.3	0	0.0
N06AB04	Citalopram	6	1.9	5	83.3
N06AB05	Paroxetine	2	0.6	0	0.0
N06AB06	Sertraline	3	0.9	1	33.3
N06AX05	Trazodone	2	0.6	1	50.0
N06AX11	Mirtazapine	6	1.9	1	16.7
N06AX16	Venlafaxine	1	0.3	0	0.0
N06BX06	Citicoline	5	1.6	4	80.0
N06D	Anti-dementia drugs	4	1.3	3	75.0
N06DA02	Donepezil	1	0.3	1	100.0
N06DA03	Rivastigmine	1	0.3	1	100.0
N06DA04	Galantamine	1	0.3	0	0.0
N06DX01	Memantine	1	0.3	1	100.0
N07CA01	Betahistine	8	2.5	4	50.0
R- Respiratory system					
R01AD05	Budesonide	1	0.3	1	100.0
S- Sensory organs					
S01EC01	Acetazolamide	1	0.3	1	100.0
S01EE01	Latanoprost	1	0.3	1	100.0
	Total active substances	103	32.4	70	68.0
	Total	318	100.0	136	42.8

n = total number of drugs recommended to withdraw, and the total number of drugs withdrawn.

%*, percentage of the drugs withdrawn compared to those recommended to be withdrawn.

Of the 45 drugs recommended to be changed in 39 patients, 11 (24.4%) were altered. The complete list of the drugs recommended to be changed and those changed during the intervention, divided according to their ATC classification, are shown in Table 3.

**TABLE 3:** Drugs recommended to be changed with the drugs changed in the pharmacological review.

Drugs recommended to change				Changed	
		n	%	n	%*
A- Alimentary tract and metabolism					
A02BC	Proton pump inhibitors	6	13.3	1	16.7
A02BC02	Pantoprazole	3	6.7	1	33.3
A02BC03	Lansoprazole	1	2.2	0	0.0
A02BC05	Esomeprazole	2	4.4	0	0.0
A06AA01	Liquid paraffin	1	2.2	1	100.0
A10BH02	Vildagliptin	1	2.2	0	0.0
B- Blood and blood forming organs					
B01A	Antithrombotic agents	5	11.1	0	0.0
B01AE07	Dabigatran etexilate	2	4.4	0	0.0
B01AF01	Rivaroxaban	3	6.7	0	0.0
C- Cardiovascular system					
C03CA01	Furosemide	1	2.2	0	0.0
C07BA06	Timolol and thiazides	1	2.2	0	0.0
C09AA02	Enalapril	1	2.2	1	100.0
C09CA	Angiotensin II receptor blockers	6	13.3	0	0.0
C09CA02	Eprosartan	1	2.2	0	0.0
C09CA04	Irbesartan	1	2.2	0	0.0
C09CA07	Telmisartan	2	4.4	1	50.0
C09CA08	Olmesartan medoxomil	2	4.4	0	0.0
C10AA	HMG CoA reductase inhibitors	7	15.6	2	28.6
C10AA01	Simvastatin	4	8.9	1	25.0
C10AA05	Atorvastatin	2	4.4	1	50.0
C10AA08	Pitavastatin	1	2.2	0	0.0
N- Nervous system					
N02AB03	Fentanyl	1	2.2	1	100.0
N02AX02	Tramadol	1	2.2	0	0.0
N02BB02	Metamizole sodium	1	2.2	0	0.0
N03AE01	Clonazepam	2	4.4	0	0.0
N05AD01	Haloperidol	1	2.2	1	100.0
N05BA	Benzodiazepine derivatives (anxiolitics)	3	6.7	2	66.7
N05BA05	Potassium clorazepate	1	2.2	1	100.0
N05BA08	Bromazepam	1	2.2	0	0.0
N05BA12	Alprazolam	1	2.2	1	100.0
N05CD11	Loprazolam	1	2.2	0	0.0
N06AB	Selective serotonin reuptake inhibitors	6	13.3	1	16.7
N06AB04	Citalopram	1	2.2	0	0.0
N06AB05	Paroxetine	4	8.9	0	0.0
N06AB10	Escitalopram	1	2.2	1	100.0
	Total active substances	29	64.4	11	37.9
	Total	45	100.0	11	24.4

n = total number of drugs recommended to change, and the total number of drugs changed.

%*, percentage of the drugs changed compared to those recommended to be changed.

Finally, of the 561 drugs recommended as adequate in 276 patients, 127 (22.6%) were withdrawn. The five most frequently recommended were quetiapine (n = 56, 10.0%), acetylsalicylic acid (n = 34, 6.1%), furosemide (n = 30, 5.3%), risperidone (n = 26, 4.6%), and trazodone (n = 26, 4.6%). From this category of drugs, the five most frequently withdrawn were quetiapine (n = 10, 7.9%), risperidone (n = 10, 7.9%), acetylsalicylic acid (n = 7, 5.6%), tramadol (n = 6, 4.8%), and pregabalin (n = 5, 4.0%). All the drugs recommended to be adequate with the drugs withdrawn, are divided according to their ATC classification, are shown in Table 4.

**TABLE 4:** Drugs recommended as adequate with the drugs withdrawn in the pharmacological review.

Drugs recommended as adequate				Withdrawn	
		n	%	n	%*
A- Alimentary tract and metabolism					
A02BC	Proton pump inhibitors	20	3.6	2	10.0
A02BC01	Omeprazole	17	3.0	1	5.9
A02BC02	Pantoprazole	1	0.2	0	0.0
A02BC03	Esomeprazole	2	0.4	1	50.0
A03FA03	Domperidone	2	0.4	0	0.0
A05AA02	Ursodeoxycholic acid	2	0.4	1	50.0
A10A	Insulins and analogues	6	1.1	1	16.7
A10AB	Insulin fast-acting	3	0.5	1	33.3
A10AE04	Insulin glargine	3	0.5	0	0.0
A10B	Blood glucose lowering drugs, excluding insulins	10	1.8	3	30.0
A10BA02	Metformin	4	0.7	1	25.0
A10BD07	Metformin and sitagliptin	1	0.2	1	100.0
A10BH	Dipeptidyl peptidase 4 inhibitors	5	0.9	1	20.0
A11CC	Vitamin D and analogues	7	1.2	1	14.3
A11DA	Vitamin B1	1	0.2	0	0.0
A12AX	Calcium with vitamin D	2	0.4	0	0.0
A12BA	Potassium	2	0.4	1	50.0
B- Blood and blood forming organs					
B01A	Antithrombotic agents	55	9.8	12	21.8
B01AA07	Acenocoumarol	2	0.4	2	100.0
B01AB05	Enoxaparin	1	0.2	1	100.0
B01AC04	Clopidogrel	9	1.6	0	0.0
B01AC06	Acetylsalicylic acid	34	6.1	7	20.6
B01AC07	Dipyridamole	1	0.2	1	100.0
B01AC18	Triflusal	1	0.2	1	100.0
B01AE07	Dabigatran etexilate	1	0.2	0	0.0
B01AF01	Rivaroxaban	1	0.2	0	0.0
B01AF02	Apixaban	3	0.5	0	0.0
B01AF03	Edoxaban	2	0.4	0	0.0
B03AA01	Ferrous glycine sulfate	11	2.0	3	27.3
B03B	Vitamin B12 and folic acid	8	1.4	4	50.0
B03BA01	Cyanocobalamin	5	0.9	1	20.0
C- Cardiovascular system					
C01AA05	Digoxin	10	1.8	1	10.0
C01BD01	Amiodarone	2	0.4	0	0.0
C03AA03	Hydrochlorothiazide	7	1.2	4	57.1
C03CA	Sulfonamides, plain	32	5.7	3	9.4
C03CA01	Furosemide	30	5.3	3	10.0
C03CA04	Torasemide	2	0.4	0	0.0
C03DA01	Spironolactone	1	0.2	0	0.0
C04AX21	Naftidrofuryl	1	0.2	0	0.0
C05AE03	Diltiazem	1	0.2	0	0.0
C07A	Beta blocking agents	14	2.5	1	7.1
C07AA06	Timolol	1	0.2	0	0.0
C07AB07	Bisoprolol	11	2.0	1	9.1
C07AG02	Carvedilol	2	0.4	0	0.0
C07BA06	Timolol and thiazides	1	0.2	0	0.0
C08CA	Dihydropyridine derivatives	5	0.9	1	20.0
C08CA01	Amlodipine	3	0.5	0	0.0
C08CA05	Nifedipine	1	0.2	1	100.0
C08CA11	Manidipine	1	0.2	0	0.0
C09AA	ACE inhibitors, plain	7	1.2	2	28.6
C09AA01	Captopril	1	0.2	0	0.0
C09AA02	Enalapril	5	0.9	2	40.0
C09AA05	Ramipril	1	0.2	0	0.0
C09BA02	Enalapril and diuretics	2	0.4	0	0.0
C09CA01	Losartan	1	0.2	0	0.0
C10A	Lipid modifying agents	26	4.6	4	15.4
C10AA01	Simvastatin	20	3.6	2	10.0
C10AA05	Atorvastatin	5	0.9	1	20.0
C10AX09	Ezetimibe	1	0.2	1	100.0
D- Dermatologicals					
D01AE14	Ciclopirox	1	0.2	0	0.0
D06AX09	Mupirocin	1	0.2	0	0.0
D11AX10	Finasteride	1	0.2	0	0.0
G- Genito urinary system and sex hormones					
G04BD12	Mirabegron	1	0.2	1	100.0
G04CA02	Tamsulosin	2	0.4	0	0.0
H- Systemic hormonal preparations					
H02AB07	Prednisone	2	0.4	1	50.0
H02AB13	Deflazacort	1	0.2	1	100.0
H03AA01	Levothyroxine sodium	4	0.7	0	0.0
J- Antiinfective for systemic use					
J01EE04	Sulfamoxole and trimethoprim	1	0.2	1	100.0
M- Musculo-skeletal system					
M04AA01	Allopurinol	6	1.1	0	0.0
N- Nervous system					
N02A	Opioids	19	3.4	7	36.8
N02AA55	Oxycodone and naloxone	1	0.2	0	0.0
N02AB03	Fentanyl	7	1.2	1	14.3
N02AJ13	Tramadol and paracetamol	2	0.4	0	0.0
N02AX02	Tramadol	9	1.6	6	66.7
N02B	Other analgesics and antipyretics	8	1.4	6	75.0
N02BB02	Metamizole sodium	5	0.9	4	80.0
N02BE01	Paracetamol	3	0.5	2	66.7
N03A	Antiepileptics	27	4.8	7	25.9
N03AA03	Primidone	1	0.2	0	0.0
N03AE01	Clonazepam	1	0.2	0	0.0
N03AX12	Gabapentin	11	2.0	2	18.2
N03AX14	Levetiracetam	1	0.2	0	0.0
N03AX16	Pregabalin	13	2.3	5	38.5
N04AA01	Trihexyphenidyl	1	0.2	0	0.0
N04B	Dopaminergic agents	3	0.5	0	0.0
N04BA02	Levodopa and decarboxylase inhibitor	1	0.2	0	0.0
N04BC05	Pramipexole	1	0.2	0	0.0
N04BD02	Rasagiline	1	0.2	0	0.0
N05A	Antipsychotics	88	15.7	23	26.1
N05AD01	Haloperidol	2	0.4	2	100.0
N05AH03	Olanzapine	1	0.2	0	0.0
N05AH04	Quetiapine	56	10.0	10	17.9
N05AL01	Sulpiride	1	0.2	0	0.0
N05AL07	Levosulpiride	1	0.2	1	100.0
N05AN01	Lithium	1	0.2	0	0.0
N05AX08	Risperidone	26	4.6	10	38.5
N05B	Anxiolytics	25	4.5	6	24.0
N05BA05	Potassium clorazepate	1	0.2	0	0.0
N05BA06	Lorazepam	20	3.6	4	20.0
N05BA08	Bromazepam	2	0.4	1	50.0
N05BA12	Alprazolam	2	0.4	1	50.0
N05C	Hypnotics and sedatives	8	1.4	2	25.0
N05CD06	Lormetazepam	3	0.5	1	33.3
N05CF02	Zolpidem	1	0.2	1	100.0
N05CM02	Clomethiazole	4	0.7	0	0.0
N06A	Antidepressants	92	16.4	15	16.3
N06AA09	Amitriptyline	2	0.4	1	50.0
N06AB04	Citalopram	15	2.7	2	13.3
N06AB05	Paroxetine	3	0.5	1	33.3
N06AB06	Sertraline	18	3.2	2	11.1
N06AX05	Trazodone	26	4.6	3	11.5
N06AX11	Mirtazapine	19	3.4	3	15.8
N06AX16	Venlafaxine	3	0.5	0	0.0
N06AX21	Duloxetine	2	0.4	0	0.0
N06AX23	Desvenlafaxine	1	0.2	0	0.0
N06AX26	Vortioxetine	3	0.5	3	100.0
N06BX06	Citicoline	1	0.2	1	100.0
N06D	Anti-dementia drugs	6	1.1	2	33.3
N06DA02	Donepezil	2	0.4	0	0.0
N06DA03	Rivastigmine	1	0.2	1	100.0
N06DX01	Memantine	3	0.5	1	33.3
N07CA01	Betahistine	2	0.4	0	0.0
R- Respiratory system					
R01AD	Corticosteroids	7	1.2	4	57.1
R01AD05	Budesonide	6	1.1	4	66.7
R01AD09	Mometasone	1	0.2	0	0.0
R03AC	Selective beta-2-adrenoreceptor agonists	2	0.4	0	0.0
R03AC12	Salmeterol	1	0.2	0	0.0
R03AC19	Olodaterol	1	0.2	0	0.0
R03BB01	Ipratropium bromide	2	0.4	1	50.0
R06A	Antihistamines for systemic use	4	0.7	3	75.0
R06AB02	Dexchlorpheniramine	1	0.2	1	100.0
R06AE07	Cetirizine	1	0.2	1	100.0
R06AX13	Loratadine	1	0.2	0	0.0
R06AX29	Bilastine	1	0.2	1	100.0
S- Sensory organs					
S01EC01	Acetazolamide	1	0.2	0	0.0
S01ED01	Timolol	2	0.4	0	0.0
S01EE01	Latanoprost	2	0.4	0	0.0
	Total active substances	116	20.7	59	50.9
	Total	561	100.0	127	22.6

n = total number of drugs recommended to adequate, and the total number of drugs withdrawn.

%*, percentage of the drugs withdrawn compared to those recommended as adequate.

In a total of 293 (60.7%) patients, between 1 and 9 drugs were withdrawn, with a mean of 2.3 (SD 1.7), and a total of 695 drugs. In spite of our recommendations for prescribed medications to be withdrawn, changed, or considered adequate, we could only record the withdrawn ones.

With respect to additional medication, in 276 (57.1%) patients, between 1 and 8 drugs were added, with a mean of 2.2 (SD 1.4), and a total of 626 drugs at the end of the intervention. The most frequently added drugs are shown in Table 5. A complete list of all the prescribed drugs that have been added are shown in Supplementary Table S1, and according to their ATC classification in Supplementary Table S2.

**TABLE 5:** List of the most frequently added drugs.

Drugs added			
ATC	Name	n	%
A11CC	Vitamin D and analogues	55	8.8
N02BE01	Paracetamol	39	6.2
N05AH04	Quetiapine	28	4.5
B03B	Vitamin B and folic acid	25	4.0
A02BC01	Omeprazole	22	3.5
C03CA01	Furosemide	21	3.4
B03AB	Iron trivalent, oral antianemic preparations	19	3.0
A12AX	Calcium, combinations with vitamin D and/or other drugs	18	2.9
A06AD	Osmotically acting laxatives	16	2.6
N06AX11	Mirtazapine	16	2.6
N05BA06	Lorazepam	15	2.4
N02BB02	Metamizole sodium	14	2.2
A10A	Insulins and analogs	11	1.8
B01AF	Direct factor Xa inhibitors	11	1.8
C07AB07	Bisoprolol	11	1.8
C08CA01	Amlodipine	11	1.8
N05AX08	Risperidone	11	1.8
N06AB06	Sertraline	11	1.8
N06AX05	Trazodone	11	1.8
B01AC06	Acetylsalicylic acid	10	1.6
D01A	Antifungals for topical use	10	1.6
C10AA01	Simvastatin	8	1.3
N02AB03	Fentanyl	8	1.3
C09CA01	Losartan	7	1.1
M05BA	Bisphosphonates	7	1.1
B01AC04	Clopidogrel	6	1.0
C09AA02	Enalapril	6	1.0
N02AX02	Tramadol	6	1.0
N05CD06	Lormetazepam	6	1.0
A10BA02	Metformin	5	0.8
C03AA03	Hydrochlorothiazide	5	0.8
N03AX16	Pregabalin	5	0.8
N05AD01	Haloperidol	5	0.8
R03BB01	Ipratropium bromide	5	0.8
C10AA05	Atorvastatin	4	0.6
N03AX14	Levetiracetam	4	0.6
N06AB04	Citalopram	4	0.6
N06DX01	Memantine	4	0.6
R06AE07	Cetirizine	4	0.6
B01AB05	Enoxaparin	3	0.5
C05AE01	Glyceryl trinitrate	3	0.5
G04CA02	Tamsulosin	3	0.5
M05BX04	Denosumab	3	0.5
N03AE01	Clonazepam	3	0.5
N05AH03	Olanzapine	3	0.5
N06AX16	Venlafaxine	3	0.5
N06DA02	Donepezil	3	0.5

n = total number of each drug added.

During the intervention, a total of 86 (17.8%) deaths were recorded. Of the 233 patients in whom the recommendations were adhered to there were 37 deaths (15.8%), and of the 165 patients who did not follow the recommendations there were 33 deaths (20.0%).

## 4 Discussion

The objective of this study was to evaluate the impact of a multidisciplinary team intervention on medication plans in nursing homes. The results showed 1,097 recommendations were provided to 82.4% of the patients. Of these proposals, 32.4% were taken up thus considerably influencing prescribing practices and accepted by the GPs. The intervention, aimed at optimizing medication management, changed the total number of prescribed medications from 3,962 to 3,893 over 1 year. A figure influenced by the fact that drugs were not only withdrawn but also added when necessary. Although such a decrease was not significant, it should be taken into account that there was a 5.9% increase in the number of prescriptions from the Catalan Health Service centers in the period 2022 compared to 2021, and 4.12% in the period 2021 compared to 2020 (Catalan Health Service, 2024). In addition, these results are similar to other studies reporting that an integrated health intervention, performed in elderly people and nursing home residents, focusing on polypharmacy and inappropriate prescribing, proved useful in improving medication use. Nevertheless, there was no statistically significant reduction in the number of prescribed medications (Wallerstedt et al., 2014; Rankin et al., 2018; San-José et al., 2021; Spinewine et al., 2021; Saeed et al., 2022; Cole et al., 2023).

### 4.1 General characterization of the institutionalized patients

A marked prevalence of HRPs and number of prescribed drugs were observed throughout the medication review in all the nursing homes. The most commonly prescribed inappropriate medications were proton pump inhibitors (PPIs), analgesics, and antipsychotics/tranquilizers, with a total of 47.8% MRPs (Anderssen-Nordahl et al., 2024). Such a finding is similar to others, as commented in a 2021 review in which the most reported inappropriate medications included psychotropic drugs, medications with anticholinergic properties, antimicrobials, nonsteroidal anti-inflammatory drugs, and PPIs (Spinewine et al., 2021). In a similar manner, it concurs with previous systematic reviews that show an overall prevalence of 43.2% PIMs, with a 49% higher prevalence estimation for European countries (Morin et al., 2016).

The elderly population often requires a greater number of medications and is more susceptible to the complexities of drug use (Ma et al., 2021). Previous studies have suggested interdisciplinary teams to target nursing homes and reduce MRPs. Despite the obvious value of medication reviews, and the recommendation of their being performed at least annually, reviews are not consistently implemented in everyday clinical settings (Kurczewska-Michalak et al., 2021). An issue that should be addressed with a multidisciplinary team approach, including a clinical pharmacologist, as has been carried out in this intervention.

### 4.2 Impact of the intervention on nursing homes

The number of drugs prescribed was not significantly different from the beginning to the end of the study. Nevertheless, the reduction in specific medications and the addition of others, point to a targeted and individualized approach. This is comparable to other studies, that describe enhancement by reducing polypharmacy and MRPs, without significance in the number of prescribed drugs after the intervention (San-José et al., 2021; Spinewine et al., 2021; Saeed et al., 2022; Cole et al., 2023).

A previous study with a control group, carried out with STOPP criteria to detect PIMs, reported that the discontinuation rate was significantly greater in the intervention group (39.7%) compared to the control (19.3%); OR (95% CI): 2.75 (1.22–6.24) (Dalleur et al., 2014). In addition, an intervention performed in nursing homes in Ireland, including a deprescribing plan guided by STOPPFrail, described a decrease in the number of chronic medications after 3 months in the intervention group compared to the control (*p* < 0.001), with a mean difference of 2.25 ± 0.54 (95% CI = 1.18–3.32). The intervention, however, presented no significant difference in mortality (*p* = 0.22) (Curtin et al., 2020), in a similar manner to other studies (Cooper et al., 2015; Spinewine et al., 2021). Our findings showed that 15.8% of the patients in whom the recommendations were followed died, compared to 20.0% in whom they were not. It should be noted, however, that the criteria of our recommendations are not exactly the same as those of the studies mentioned. Furthermore, some articles have described a lower risk of death (Kua et al., 2019; Sluggett et al., 2022). A retrospective cohort study in Australia examining medication reviews in nursing homes showed a 4.4% lower mortality risk (95% CI = 0.02–8.60, *p* = 0.048) over 12 months (Sluggett et al., 2022). In a systematic review and 2019 meta-analysis of randomized controlled trials in nursing homes, when a subgroup analysis was performed in the medication review, the deprescribing interventions reduced mortality by 26% (OR 0.74, 95% CI = 0.65–0.84) (Kua et al., 2019).

Our study revealed a significant impact on medication with changes, and in 58.5% of the patients who received recommendations, they were followed. Notably, antipsychotics, antidepressants, benzodiazepines, statins, and diuretics were the most frequently withdrawn drugs, indicating a concerted effort to reduce MRPs. A finding similar to other studies, such as an observational before-after intervention where the medications withdrawn included antipsychotics, antidepressants, sedatives, and diuretics (Fog et al., 2017). In a retrospective cohort study conducted in Madrid, Spain, pharmacist-led medication reviews identified an average of 4.85 (SD 3.33) MRPs per patient, with 86.73% of the proposed changes being accepted. This intervention reduced the average number of medications by 2.09 (95% CI: 1.98–2.21; *P*< .001) per patient (Peral Bolaños et al., 2024). Similarly, another retrospective observational multicentric pre-post study assessed the impact of clinical pharmacist medication reviews on the quality of pharmacotherapy in primary care psychogeriatric patients with excessive polypharmacy. The study found that clinical pharmacists proposed 374 interventions in psychopharmacotherapy, with GPs accepting 45.2% of them. This acceptance led to a 7.5% reduction in the total number of medications (*p* < 0.05) and a 21.8% reduction in the number of prescribed potentially inappropriate medications (PIMs) (*p* < 0.05), among other outcomes (Stuhec and Zorjan, 2022).

Whilst there was no specific intervention in the use of absorbent pads during this study, we observed a 9.5% increase, likewise with the optimization of psycholeptic drugs. Previous studies in patients with dementia have shown that the administration of antipsychotics increases mortality (Connors et al., 2016; Schwertner et al., 2019), and a higher risk of falls in the elderly with antipsychotic drugs, among others (Zhou et al., 2022). A recent cohort study based on electronic records in the United Kingdom demonstrated that the use of antipsychotics in patients with dementia was associated with greater risk of stroke, venous thromboembolism, myocardial infarction, heart failure, fracture, pneumonia, and acute kidney injury. Choosing the appropriate antipsychotic, determining dosage, and managing treatment duration are essential factors to prevent adverse reactions linked to its usage (Mok et al., 2024). It is also crucial to carry out specific interventions in institutionalized patients due to the considerable misuse of psycholeptic drugs. These observations could be a focal point for proposed action in future studies.

### 4.3 A multidisciplinary team approach

The multidisciplinary approach is a recurring theme, underscoring the importance of collaborative decision-making. Collaborative efforts within such teams play a key role and lead to optimal individualized medication management for nursing home residents (Fog et al., 2017; Disalvo et al., 2020; Song et al., 2023).

A qualitative study concerning the barriers and facilitators that affect the process of conducting medication reviews identified organizational hurdles, time constraints, and communication challenges among healthcare professionals as barriers. Key facilitators included improved communication channels, collaboration within multidisciplinary teams, and resident and family engagement in decision-making. The study provides valuable insights into the complexities of medication management in this vulnerable population (Wouters et al., 2019). All these aspects were included in our intervention considering the limitations of the lockdown period.

A systematic review investigating strategies to manage polypharmacy highlighted the importance of multifaceted interventions, including patient-centered approaches, interdisciplinary collaboration, and technology-driven solutions. It emphasized the role of education and awareness programs targeting healthcare professionals and older adults. Medication reviews, deprescribing efforts, and the integration of technology, such as clinical decision support systems, emerge as promising avenues to optimize medication regimens and enhance patient safety (Kurczewska-Michalak et al., 2021).

Findings from our study suggest that the intervention, guided by comprehensive recommendations, with different proposals, individualized improvement plans, and changes in data registration, holds promise for optimizing medication regimens in nursing homes. Our results should encourage interventions that prioritize the individual needs and preferences of the residents thus potentially improving adherence and overall health outcomes. Nevertheless, challenges and considerations should be recognized. Whilst patient quality of life in nursing homes has been described in previous reviews and interventions with control groups, differences in health-related quality of life have not been described (Cooper et al., 2015; Curtin et al., 2020; Cole et al., 2023). The logistical aspects of coordinating a multidisciplinary team, ensuring effective communication, and addressing potential conflicts in treatment plans require careful management. We believe this could be managed by incorporating a clinical pharmacologist, as shown in Figure 1, to ensure at least one annual pharmacological review in nursing homes.

## 5 Strengths and limitations

Our study presents multiple strengths and limitations. The intervention was carried out at the beginning of the COVID-19 pandemic and with the declaration of a state of alarm by the Spanish government (BOE-A-2020-3692, 2020). This entailed inherent difficulties, such as having appointments with patients admitted to nursing homes, which hindered the actual intervention and patient follow-up. To the best our knowledge, however, this is the first study to analyze the impact of an intervention on nursing homes in Catalonia after reviewing prescribed medications and individually giving recommendations. Data from five different nursing homes were gathered. The medical review was performed by a clinical pharmacologist, with the possibility of changing prescriptions when needed and providing individual recommendations. The availability of a common computerized data system helped review the prescription registry and made coordination possible among nursing homes, primary care, and hospital care. It was an advantage that this project included primary care professionals, nursing home staff, physicians specialized in geriatrics, clinical pharmacology, and a clinical pharmacist, thus creating a multidisciplinary team, with an agreed final decision. A project that allows us to form new proposals to improve future interventions.

With respect to limitations, the extrapolation of our findings to other regions or countries should be performed with caution since the intervention was conducted in one urban area. There was no sample size calculation since all the patients from the nursing homes, where the intervention was conducted were included. Nevertheless, as the intervention covered 22.3% of the population in the northern area of Barcelona, Catalonia, it may be representative of areas with a similar socioeconomic level. The intervention was carried out in routine clinical practice, some information therefore is lacking, such as non-pharmacological treatments, non-registered treatments, or those not financed by the public health system. Neither are there data on drug adherence as the patients’ clinical records are intended for assistance and not research. The different outcomes between the nursing homes could not be reviewed since the study was not designed for this and it was not the main goal of the intervention. Furthermore, the correlation between drugs and death was not adjusted for age or comorbidities. Since the intervention was performed during the COVID-19 pandemic, the patients’ safety was prioritized, and the complex situation meant there was no adequate optimization of psychotropic drugs. A similar study with a control group, and out of the pandemic context, should be repeated in the elderly in different regions to confirm these results.

## 6 Conclusion

In conclusion, many recommendations were made confirming the increasing incidence of polypharmacy and the need for standardized interventions targeting nursing homes. They could help reduce MRPs and the number of prescribed drugs, with the aim of safer drug use. The favorable outcomes of this intervention highlight the importance of collaborative healthcare models in optimizing medication practices and set a precedence for future innovations in geriatric care. A multidisciplinary team providing a patient-centered approach, interdisciplinary collaboration including a clinical pharmacologist, and technology-driven solutions, should help reduce MRPs and polypharmacy.

## Data Availability

The original contributions presented in the study are included in the article/Supplementary Material, further inquiries can be directed to the corresponding author.
